# Comprehensive analysis of volatile compounds in cold‐pressed safflower seed oil from Xinjiang, China

**DOI:** 10.1002/fsn3.1369

**Published:** 2019-12-27

**Authors:** Lin Wang, Zhuo Chen, Bo Han, Wenxia Wu, Qiaoling Zhao, Changqing Wei, Wenyu Liu

**Affiliations:** ^1^ School of Food Science and Technology/Key Laboratory of Xinjiang Phytomedicine Resource and Utilization Ministry of Education Shihezi University Shihezi China; ^2^ School of Pharmacy/Key Laboratory of Xinjiang Phytomedicine Resource and Utilization of Ministry of Education Shihezi University Shihezi China; ^3^ Post‐Doctoral Research Station of Xinjiang Sailimu Modern Agriculture Co. Bole China

**Keywords:** headspace solid‐phase micro‐extraction gas chromatography coupled with mass spectrometry, principal component analysis, relative odor activity value, safflower seed oil

## Abstract

Three varieties of safflower seed oil (SSO) from Xinjiang Autonomous Region, China, were analyzed by headspace solid‐phase micro‐extraction gas chromatography coupled with mass spectrometry (HS‐SPME‐GC‐MS) to reveal volatile components. Overall, 67 volatile components were determined and four compounds including isoamyl alcohol, caproic acid, n‐pentanal, and heptanal were newly identified in SSO as aroma‐active components. Meanwhile, 16 compounds were selected by relative odor activity value (ROAV) to evaluate contributions of single compounds to the overall odor (ROAV > 1), in which nonanal, (Z)‐6‐nonenal, and (E)‐2,4‐decadienal were the top three contributed substances (ROAV > 70). The sensory panel was described as eight definition terms (grassy, fruity, almond, mushroom, fatty, sweet, paddy, and overall fragrance). Principal component analysis (PCA) revealed a significant separation of three cultivars with the first principal component (PC‐1) and the second principal component (PC‐2) expressing 73.9% and 23.1%, respectively. Both PCA and ROAV allowed identifying the compounds positively correlated to sensory evaluation.

## Graphical abstract

Headspace solid‐phase micro‐extraction gas chromatography coupled with mass spectrometry combined with relative odor activity value (ROAV) and sensory analysis to study the cultivated varieties' contribution in flavor character of safflower seed oil. Four compounds including isoamyl alcohol, caproic acid, n‐pentanal, and heptanal were newly identified, and 16 compounds were selected to evaluate contributions of single compounds to the overall odor (ROAV > 1). PCA revealed a clear discrimination.

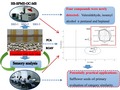

## 1 INTRODUCTION

Safflower (*Carthamus tinctorius* L.) belonging to the *Compositae* family is widely cultivated in China, India, the United States, and Mexico as oilseed crop (Mihaela, Josef, Monica, & Rudolf, 2013). The root system of safflower makes it an ideal crop for arid agricultural land owing to its ability to withstand (de Oliveira et al., 2018). The core area of the arid region in Central Asia and Xinjiang Autonomous Region, China, has a special climate with great temperature difference between day and night, drought, and ample sunshine; thus, it is one of the suitable safflower‐growing regions in the world (Yao et al., 2019). The annual yield of Chinese safflower seeds has gone up to about 360,00 thousand tonnes, and northwest of Xinjiang Autonomous Region is the top growing area with a share of 80% (Hussain, Lyra, Farooq, Nikoloudakis, & Khalid, 2016; Lu et al., 2013). Additionally, SSO in this area also has a highest content of linoleic acid (85.04%) compared with other districts in China (Han, Cheng, Zhang, & Bi, 2009). The three most popular cultivars of safflower (Tacheng HH‐4, Bole HH‐1, and Changji HH‐2) devoted to SSO were collected in different locations from Xinjiang, China (Liu et al., 2009).

Safflower seeds contain 38%–48% oil, which has 6%–8% palmitic (C16:0), 2%–3% stearic (C18:0), 16%–20% oleic (C18:1), 0.2%–0.4% linolenic (C18:3), and 71%–75% linoleic acids (C18:2) in its composition (Liu, Guan, & Yang, 2016). SSO has been used to reduce the cholesterol level in the blood and improve glycemia in clinical trials owing to its great quantity of polyunsaturated fatty acids, especially oleic and linoleic acids (Fernandes, Tache, Klingel, Leri, & Mutch, 2018; Tso, Caldwell, Lee, Boivin, & Michele, 2012). Zhang et al. determined that the presence of phenolic compounds and flavonoids in SSO also has been observed and makes safflower seeds became a focus in scientific literature. There are some studies indicating that bone loss and marrow adiposity could be prevented by phenolic compounds, which also has shown effect to ameliorate the progress of atherosclerosis and inhibit vascular distensibility (Zhang, Liu, Pu, Sun, & Zhao, 2017). Besides knowledge of their composition and nutritional quality, since cold press oils are virgin products, their aromatic profiles and sensory properties are also essential components for consumer acceptance (Dun et al., 2018).

Flavor is considered as the key driver of consumer's appreciation and is closely related to both qualitative and quantitative composition of volatile and nonvolatile compounds (Sabatini & Marsilio, 2008). Romero, García‐González, Aparicio‐Ruiz, and Morales (2015) showed that high potency of some volatile compounds were responsible for sensory defects and different mixing ratios generated different sensations. Erten and Cadwallader (2017) indicated that yield area, processing technology, and environment could be known by analyzing aroma profiling of oil‐roasted almonds. In fact, although many complete characterization of sensory profiles have been reported for other oils such as olive oil, peanut oil, and palm kernel oil (Lim et al., 2017; Marina, Che Man, & Amin, 2010; Morales, Luna, & Aparicio, 2000), few investigation of SSO relating sensory attributes to volatile components was carried out, especially the SSO from Xinjiang Autonomous Region, China. Moreover, although SSO from different sources has been characterized for their sensory properties (Baiano, Terracone, Viggiani, & Nobile, 2013), few cultivars have received in‐depth investigation of their volatile compounds. To the best of our knowledge, there is only one literature published concerning the sensation description and volatile compounds of SSO from Xinjiang Autonomous Region, China, in which only one type had been detected (Aydeniz, Güneşer, & Yılmaz, 2014). Therefore, it is necessary to conduct a study for remedying this aspect.

Currently, several techniques have continuously investigated to achieve quick and efficient extracting for volatile components including supercritical fluid extraction (SFE), enzymatic oil extraction, and pressurized liquid extraction (Conte et al., 2016; Gibbins, Aksoy, & Ustun, 2012; Han et al., 2009; Hu et al., 2012), among them, headspace solid‐phase micro‐extraction (HS‐SPME) is a common extraction method beneficial for isolation and preconcentration of volatiles with advantages of fast, simple, sensitive, and no solvent, prior to gas chromatographic analysis (Mesquita et al., 2017). However, high content of volatile compounds not necessarily has a high contribution value; thus, a parameter named “relative odor activity value (ROAV),” which was frequently combined with electronic nose and GC‐MS technology to assign the key odor compounds, was widely applied to evaluate the contributions of the single compound to the overall odor (Multari, Vall, Yang, & Suomela, 2018). Sun et al. related key odor compound data of ROAV with principal component analysis (PCA) to reveal a clear classification of aroma profiling of star anise, and the cluster analysis showed a clear consistency with ROAV analysis results (Sun, Chen, Li, Liu, & Zhao, 2014). It is known that in general, the perceived odors in foods are the result of a mixture of odorant; hence, a multivariate analysis is suited to explore the relationship between sensory attributes and volatile compounds (Wang, Zou, Shi, & Shi, 2018). Despite extensive studies on other plants' oil (Sun et al., 2014), no comparative study using ROAV has been published regarding aroma contributors of SSO.

The objectives of the present study were as follows: (a) to study the volatile composition and sensory attributes of different cultivated samples of SSO in Xinjiang Autonomous Region, China, using headspace solid‐phase micro‐extraction gas chromatography coupled with mass spectrometry (HS‐SPME‐GC‐MS) analysis, respectively, (b) to elucidate the relationship between volatile compounds and sensory attributes by ROAV and identify the compounds that could potentially contribute to odor perception, and (c) to apply PCA for visualizing their cluster trends of SSO of various origins characterized by their volatile profiles.

## 2 MATERIALS AND METHODS

### 2.1 Samples

Safflower seeds from the three most popular cultivars (Tacheng HH‐4, Bole HH‐1, and Changji HH‐2) were harvested in early September 2017 from the Xinjiang Autonomous Region, China. The safflower seeds were cold‐pressed by oil press (ZY‐22A) at temperature of 45°C. The safflower seed oils extracted from the seeds of HH‐1, HH‐2, and HH‐4 safflower varieties were called SSO‐1, SSO‐2, and SSO‐3, respectively. All other chemicals and standards were analytical grade and purchased from Zhengzhou Wanbo Chemical Products Co., Ltd.

### 2.2 Sensory and quality analysis

The sensory profile is evaluated by well‐trained panel of 10 personnel (five females and five males without smoking history) between the age‐group of 25–50. They were recruited because of their major role in the implementation of the method for the sensory analysis of SSO and professionally trained by Food College of Shihezi University in accordance with ISO standards. Analysis of safflower seed oil samples is according to the requirements of GB/T22465‐2008. Initial draft of descriptors through current references was meticulously discussed under suitable laboratory environment. In total, there were eight descriptions, including grassy, fruity, almond, mushroom, fatty, sweet, paddy, and total acceptance. After training and once the judges agreed to be familiar with the new set of descriptors, the panel was used for this study. Oil samples were served in cups coded with a 3‐digit random number in a balanced order for presentation. Each sample was re‐evaluated three times, and the code was rescrambled each time. Samples were scored using a ten‐point scale ranging from 1.0 (very slight) to 10.0 (very intense), at intervals of 1.0.

### 2.3 HS‐SPME‐GC‐MS analysis

Volatile compounds were analyzed by HS‐SPME‐GC‐MS referencing the procedure described in a previous work (Durant et al., 2014; Wei, Zhou, Han, Chen, & Liu, 2018). Ten gram of SSO samples was inserted in a 125‐ml SPME vial. Then, the vial was closed with a Teflon/silicone septum. The fiber coated with polydimethylsiloxane/polydimethylsiloxane/divinylbenzene (PDMS/PDMS/DVB) 50/30 μm fiber (model 57348‐U, Supelco Inc.) was inserted through the septum and exposed to the headspace of the SPME vial after conditioning for 2 hr at 250°C. Then, extraction was carried out at 60°C for 15 min.

Volatile compounds adsorbed by the SPME fiber were desorbed in the injector port of the GC for 3 min at 250°C and identified by GC‐MS (Trace 2000 DSQ, Finnigan Co.). Separation was achieved on a DB‐WAX capillary column (30 m × 0.25 mm × 0.25 μm film thickness, Supelco Co.). The carrier gas was high‐purity helium at a constant flow of 0.8 ml/min with the injector temperature of 250°C. Oven temperature programming was as follows: initiated at 40°C, held for 3 min, then rose at 6°C/min to 120°C, and then 120–240°C at 10°C/min, and held there for 8 min. The temperature of transfer line is 250°C. The mass spectra were obtained using a mass selective detector by electronic impact at 70 eV with a scan range from m/z 33 to 373, emission current 200 μA, and ion source temperature 200°C.

Compound identification was based on mass spectral data of samples with the standard NIST and Wiley Library and with the comparison of retention indices (RI; positive and negative matching >800). Meanwhile, the relative content of each volatile compound was calculated by a ratio of the peak area of each component to total area of peaks in typical GC‐MS total ion chromatograms (TIC).

### 2.4 ROAV determination

The relative odor activity value (ROAV) is used to evaluate the contribution of individual compounds to the overall aroma (Cui, Liu, & Li, 2010). The formula of ROAVA is defined as the equation:

$${\text{ROAV}}_{A}=\frac{C_{A}}{T_{A}}\times \frac{T_{\text{MAX}}}{C_{\text{MAX}}}\times 100.$$

where *C*_A_ is the concentration of the compound A in the sample and *T*_A_ is its odor detection threshold concentration found in the literature. *T*_MAX_ and *C*_MAX_ are the maximum of *C*_A_/*T*_A_ among all the compounds in the sample.

Relative odor activity value ranges from 0 to 100. Volatile compound with ROAV ≥ 1 is considered as key odor compounds, of which >0.1 and smaller than 1 play a embellish role in aroma.

### 2.5 Multivariate statistical analysis

Cluster analysis (CA) is the unsupervised classification of patterns (feature vectors) into groups, so that individuals within the same group are more similar to each other than those belonging to different groups (Cebi, Dogan, Develioglu, Yayla, & Sagdic, 2017). Principal component analysis (PCA) and hierarchical cluster analysis (HCA) were carried out on the entire data obtained from the HS‐SPME‐GC‐MS in an attempt to identify the similarity of the samples. HCA dendrogram was calculated by Euclidean distance with Ward's method. All these analyses were made by means of the SPSS 8.0 package.

## 3 RESULTS

### 3.1 HS‐SPME‐GC‐MS analysis of volatile compounds

#### 3.1.1 Semi‐identification of volatile compounds

The HS‐SPME‐GC‐MS system was used to analyze and quantify the volatile components of SSO. A total of 67 volatile compounds were profiled in all SSO samples (Table 1), which were classified into 9 chemical families. Forty‐four, 41, and 47 volatile compounds were detected in different SSO samples of SSO‐1, SSO‐2, and SSO‐3, respectively. It is worth mentioning that some volatile compounds were only available in specific sample; for example, 1‐octene, 2‐octene, nonane, 3‐pentanol, butyl formate, terpinene, 3‐hydroxy‐2‐butanone, dimethyl sulfoxide, and 2,5‐octanedione were the only volatile compounds detected in SSO‐1, while SSO‐2 and SSO‐3 had its own unique volatile compounds in a number of 8 (o‐xylene, crotononitrile, myrcene, 1‐nonanol, hexyl hexanoate, (Z)‐2‐octenol, vinyl hexanoate, and (Z)‐2,4‐decadienal) and 7 (toluene, meta‐xylene, butyl acrylate, 2‐octanone, 3‐ethyl‐2‐methyl‐1,3‐hexadiene, tetradecane, and butyric acid).

**Table 1:** Volatile compounds identified by HS‐SPME‐GC‐MS in safflower seed oil from different regions

No.	ID^a^	Retention time^b^ (min)	Compound	Relative content (%)		
				SSO‐1	SSO‐2	SSO‐3
1	A,B,C	3.081	1‐Octene	1.285	0.000	0.000
2	A,B,C	3.269	2‐Octene	15.566	0.000	0.000
3	A,B,C	3.809	Nonane	0.577	0.000	0.000
4	A,B,C	4.063	3‐Methyl butanal	1.153	0.368	0.539
5	B,C	5.038	N‐butyl ether	0.000	0.125	0.307
6	A,B,C	5.270	N‐valeraldehyde^c^	1.416	0.617	0.700
7	C	6.231	2‐Pinene	1.907	5.160	1.459
8	B,C	6.674	Toluene	0.000	0.000	0.659
9	C	7.618	Butyl acetate	0.335	0.000	0.142
10	C	7.816	N‐hexanal	23.243	10.605	20.370
11	A,B,C	8.362	Undecane	0.293	0.000	0.442
12	C	8.432	Beta‐pinene	0.000	0.441	0.983
13	A,B,C	8.635	3‐Pentanol	0.207	0.000	0.000
14	C	8.85	Sabenene	0.568	0.827	1.326
15	C	9.396	O‐xylene	0.000	0.052	0.000
16	C	9.399	Paraxylene	0.140	0.000	0.746
17	C	9.4	Meta‐xylene	0.000	0.000	1.317
18	A,B,C	9.519	Butyl formate	1.006	0.000	0.000
19	A,C	9.754	Crotononitrile	0.000	3.122	0.000
20	A,C	10.137	Myrcene	0.000	0.962	0.000
21	C	10.479	Methacrylonitrile	1.220	7.335	0.000
22	B,C	10.101	Terpinene	0.752	0.000	0.000
23	A,B,C	10.493	Butyl acrylate	0.000	0.000	0.701
24	A,B,C	10.706	Heptanal^c^	0.712	0.195	0.000
25	A,B,C	10.804	Methyl caproate	0.000	0.060	0.124
26	B,C	11.064	Limonene	0.000	1.418	2.082
27	C	11.32	3‐Isopropyl‐6‐methylene‐1‐cyclohexene	0.562	0.000	0.154
28	A,B,C	11.473	Isoamyl alcohol^c^	1.537	0.051	0.651
29	A,B,C	11.589	Trans‐2‐hexenal	1.224	0.304	0.854
30	C	12.063	2‐N‐pentylfuran	1.426	2.484	4.647
31	B,C	12.708	N‐pentanol	1.409	2.387	3.518
32	B,C	13.099	P‐Isopropyltoluene	1.296	3.165	0.799
33	A,B,C	13.467	3‐Hydroxy‐2‐butanone	0.518	0.000	0.000
34	B,C	13.522	2‐Octanone	0.000	0.000	0.588
35	A,B,C	13.619	Octanal	0.000	0.322	0.638
36	A,C	14.43	Trans‐2‐heptenal	8.755	2.368	2.694
37	A,B,C	15.399	N‐hexanol	4.900	29.929	23.093
38	A,B,C	15.913	Nonanal	1.269	0.531	1.510
39	A,C	16.167	3‐Octene‐2‐one	0.000	0.811	1.722
40	C	16.342	3‐Ethyl‐2‐methyl‐1,3‐exadiene	0.000	0.000	0.723
41	A,B,C	16.56	2‐Octenal	0.973	1.962	2.650
42	B,C	16.835	Tetradecene	0.000	0.000	0.210
43	A,B,C	16.917	Acetic acid	10.149	0.785	1.803
44	C	17.011	1‐Octen‐3‐ol	0.000	2.238	4.285
45	B,C	17.239	1‐Nonanol	0.000	0.884	0.000
46	A,B,C	17.725	Decanal	0.164	0.048	0.238
47	B,C	18.107	Benzaldehyde	2.218	0.000	2.431
48	A,B,C	18.288	Cis‐6‐Nonenal	0.939	0.000	0.940
49	C	18.41	1‐Hexadecene	1.156	1.043	1.295
50	C	18.863	Dimethyl sulfoxide	0.784	0.000	0.000
51	B,C	19.263	Caryophyllene	0.218	0.119	0.234
52	A,B,C	19.342	Hexyl hexanoate	0.000	0.323	0.000
53	C	19.355	γ‐valerolactone	0.134	0.000	0.103
54	A,B,C	19.506	Trans‐2‐octenol	0.000	2.545	0.000
55	A,C	19.604	1,4‐Butanolide	0.781	0.000	0.493
56	A,B,C	19.753	Butyric acid	0.000	0.000	0.735
57	B,C	19.782	Phenylacetaldehyde	0.804	0.519	0.000
58	C	20.086	2,5‐Octanedione	0.195	0.000	0.000
59	A,B,C	20.089	Vinyl hexanoate	0.000	0.874	0.000
60	A,B,C	20.335	Isovaleric acid	1.571	0.000	2.127
61	C	20.561	γ‐caprolactone	0.252	0.555	0.488
62	A,C	20.874	2(3H)‐furan ketone	0.344	0.125	0.334
63	A,B,C	21.044	N‐pentanoic acid	0.000	0.789	0.854
64	B,C	21.837	Trans‐2,4‐decadienal	0.000	0.247	0.000
65	A,B,C	21.839	2,4‐Decadienal	0.258	0.000	0.449
66	A,B,C	22.556	Caproic acid^c^	0.955	7.879	4.576
67	B,C	23.181	Phenyl ethyl alcohol	0.782	0.301	0.876

a ID: The identification was indicated by the following symbols, A = mass spectrum and RI agree with that of the authentic compound run under similar GC‐MS conditions, B = mass spectrum and LRI agree with literature data: (1) Multari et al. (2018), (2) Feng et al. (2017), (3) Cui et al. (2010), and (4) Gao et al. (2014). C = tentative identification based on interpretation of mass spectrum and comparison with similar compounds.

b RI: retention indices calculated on DB‐WAX column. Compound: compounds with positive and negative matching >800. RI and Relative content: the mean values of parallel experiment.

c Compound: Compounds were newly identified in SSO as aroma‐active odorant.

#### 3.1.2 Overall volatile compounds profile analysis

**Figure 1:**
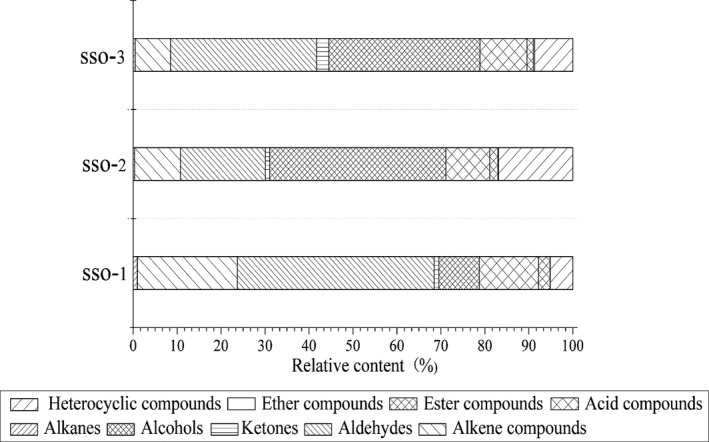
Relative content (%) of the volatile compounds from different regions of safflower seed oil

As shown in Table 1, a significant disparity was found on the grouped chemical families, the major chemical families that were identified in three oil samples were dominated by aldehydes (19.28%–44.75%) and alcohols (9.16%–40.13%), especially hexanal and n‐hexyl alcohol at a total level of 28.14%–43.46%, while minimum content was ether compounds (0%–0.33%). There was also a wide variety in the relative content of the volatile constituents among different SSOs. Hexanal accounted for 23.24% in SSO‐1 oil sample, which was slightly higher than that of SSO‐3 (20.37%) but approximately 3 times higher than that of SSO‐2 (10.60%). The compound of n‐hexyl alcohol in SSO‐1 (4.90%) is about one‐sixth of SSO‐2 (29.93%), which is about one‐fifth of SSO‐3 (23.09%). SSO‐1 performed a largest concentration values in alkanes, aldehydes, alkene compounds, acid compounds, and ester compounds with the richest in aldehydes and alkene compounds (44.75% and 22.78%, respectively) while none of ether compounds while SSO‐1 had no ether compounds. The relative content of alcohols and heterocyclic compounds of SSO‐2 (40.13% and 16.92%, respectively) was higher than other varieties; SSO‐3 detected the highest proportion of ketones with 2.80% (Figure 1).

### 3.2 Determination of main odor‐active compounds

Relative odor activity value was a new parameter, considering odor threshold, used to assess the contribution of the main odor‐active compounds to SSO. The volatiles selected by sensory thresholds and individual proportion with ROAV > 1 are all shown in Table 2. There are a total of 16 key odor compounds, including 12 aldehydes, two alcohols, one alkene, and one heterocyclic compound, of which SSO‐1, SSO‐2, and SSO‐3 only contained 12, 14, and 14 key odor‐active substances, respectively. Within the group of aldehyde, (Z)‐6‐nonenal and 2,4‐decadienal perform a relatively significant odor activity in other two samples (ROAV = 100.00–85.90, 31.39–46.76) yet a modification to SSO‐2 (ROAV = 3.12, 1.37). (E)‐2,4‐decadienal is an isomeric form for 2,4‐decadienal, which only existed in SSO‐2 with ROAV of 72.98. 1‐Octen‐3‐ol had odor threshold of 1 ppb and, being a key odorant with ROAV > 30, was only contribute to the aroma of SSO‐2 and SSO‐3 (46.37 and 31.22, ROAVs, respectively). In all three SSO samples, nonanal constituted an abundant compound, obtained the ROAV of 98.34–100.00, and gave the highest contribution to the odor with fragrance of almond and floral.

**Table 2:** Main odor‐active compounds (ROAV ≥ 1) in different varieties of safflower seed oil

No.	Compound^a^	ROAV^b^			Aroma threshold^c^ (μg/L)	Odor descriptor^d^
		SSO‐1	SSO‐2	SSO‐3		
1	N‐pentanal^e^	1.34	1.42	0.57	9.00	Fatty, Woody
2	Hexanal	39.59	43.95	29.68	5.00	Fatty, Vanilla
3	Heptanal^e^	2.17	1.45	0.00	2.80	Fried seed, Almond
4	Limonene	0.00	2.94	1.52	10.00	Fruity, Orange
5	Trans‐2‐hexenal	34.73	21.02	20.74	0.30	Sweet, Vanilla
6	2‐N‐pentylfuran	2.09	8.88	5.84	5.80	Paddy, Green beans
7	Octanal	0.00	9.53	6.64	0.70	Vanilla, Orange
8	Trans‐2‐heptenal	5.52	3.63	1.45	13.50	Fatty, Grassy
9	N‐hexanol	0.17	2.48	0.67	250.00	Almond, Grassy
10	Nonanal	98.23	100.00	100.00	0.10	Grassy, Almond
11	2‐Octenal	8.28	40.66	19.30	1.00	Mushroom, Grease
12	1‐Octen‐3‐ol	0.00	46.37	31.22	1.00	Mushroom, Sweet
13	Decanal	13.97	9.90	17.34	0.10	Grassy
14	(Z)‐6‐nonenal	100.0	0.00	85.59	0.08	Grassy, Fatty
15	Trans‐2,4‐decadienal	0.00	72.98	0.00	0.07	Grassy, Fatty
16	2,4‐Decadienal	31.39	0.00	46.76	0.07	Grassy, Fatty

a Volatile compounds identified in HS‐SPME‐GC‐MS.

b ROAV was calculated by formula ${\text{ROAV}}_{A}=\frac{C_{A}}{T_{A}}\times \frac{T_{\text{MAX}}}{C_{\text{MAX}}}\times 100.$

c Aroma threshold was determined in oil by according to literature: (1) Multari et al. (2018), (2) Feng et al. (2017), (3) Cui et al. (2010), and (4) Gao et al. (2014).

d Odor descriptor was obtained according to the literature: (1) Erten and Cadwallader (2017), (2) Romero et al. (2015), and (3) López‐López, Sánchez‐Gómez, Montaño, Cortés‐Delgado, & Garrido‐Fernández, (2019).

e Compound: Compounds were newly identified in SSO as key aroma‐active compounds.

### 3.3 Sensory evaluation

**Figure 2:**
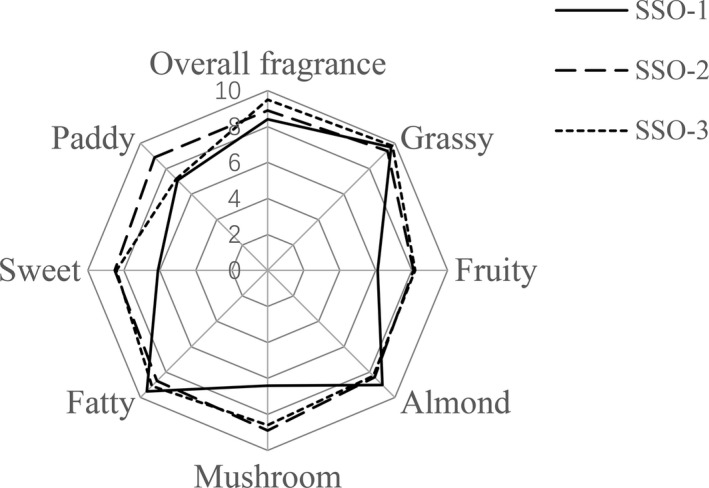
Sensory panel descriptive analysis average scores from different regions of safflower seed oil

The average scores of sensory descriptive in the spider web form for SSO are shown in Figure 2. Eight panel sensory terms were used to assess flavor characteristics including grassy, fruity, almond, mushroom, fatty, sweet, paddy, and overall fragrance. As shown in Table 3, by comparing all average score for the sensory evaluation among three SSO samples of different varieties, “grassy,” “almond,” and “fatty” descriptors were of comparable intensity (and all gained high scores) while the remaining odor profiles were differed significantly (*p* < .05). It is worth mentioning that despite species different, all SSO samples represented characteristically “grassy” aroma in a highest level (score > 9.7). Besides, SSO‐1 was also abundant in intense of “almond” and “fatty” with a score of 9.0 and 9.5, while “mushroom” (8.9) and “paddy” (8.9) were highest in the SSO‐2; SSO‐3 was characterized by “fatty” aroma (score = 9.1). It is noteworthy that the terms of SSO‐2 and SSO‐3 showed obviously higher olfaction scores in “fruity” (8.2), “mushroom” (8.6), and “sweet” aroma (8.4) than SSO‐1. Sensory odor map also indicated that except paddy term, all of the sensory scores gave similar intensity values for SSO‐2 and SSO‐3. SSO‐3 represented a best level with score 9.5 in all fragrance.

**Table 3:** Multiple comparison analysis of sensory characterization from different varieties of safflower seed oil

Sample	Overall fragrance							
	Grassy	Fruity	Almond	Mushroom	Fatty	Sweet	Paddy	Overall fragrance
SSO‐1	9.7^e^	6.1^e^	9.0^f^	6.4^e^	9.5^g^	6.1^e^	7.1^e^	8.4^e^
SSO‐2	9.4^e^	8.1^f^	8.4^e^	8.9^f^	8.7^e^	8.5^f^	8.9^f^	8.9^f^
SSO‐3	9.8^e^	8.2^f^	8.3^e^	8.6^f^	9.1^f^	8.4^f^	7.2^e^	9.5^g^
*p* *	.7	>.016	>.049	>.041	.2	>.015	.5	.2

* *p*: Values with unlike letters (e–g) differ significantly (*p* ≤ .05).

### 3.4 Multivariate statistical analysis

**Figure 3:**
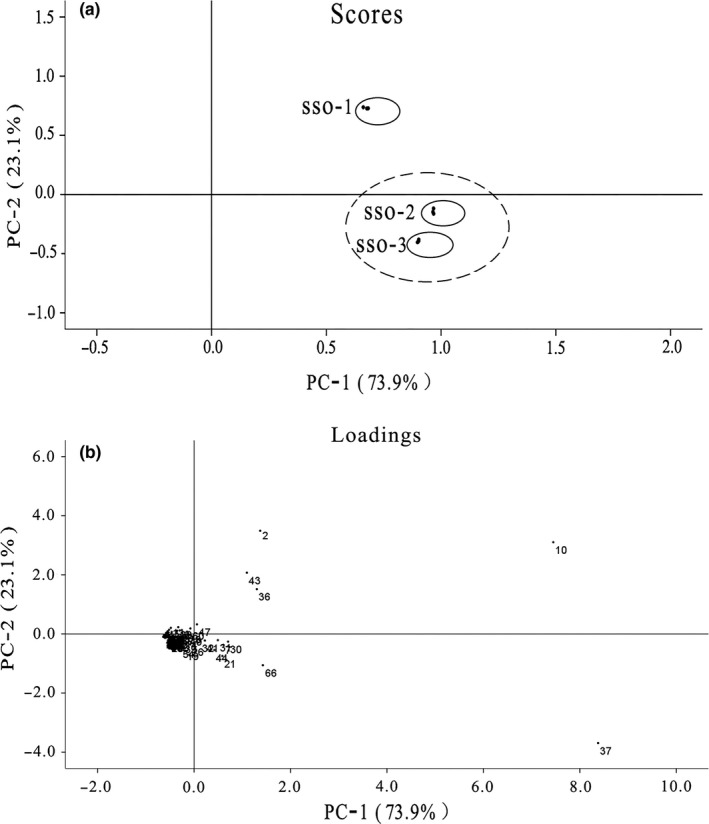
PCA of volatile compounds extracted by HS‐SPME‐GC‐MS. (a) Score plot of PC‐1 versus PC‐2 scores. (b) Loading plot for PC‐1 and PC‐2 contributing volatile and their assignments. Total variance PC‐1 versus PC‐2 is 97.0%

**Figure 4:**
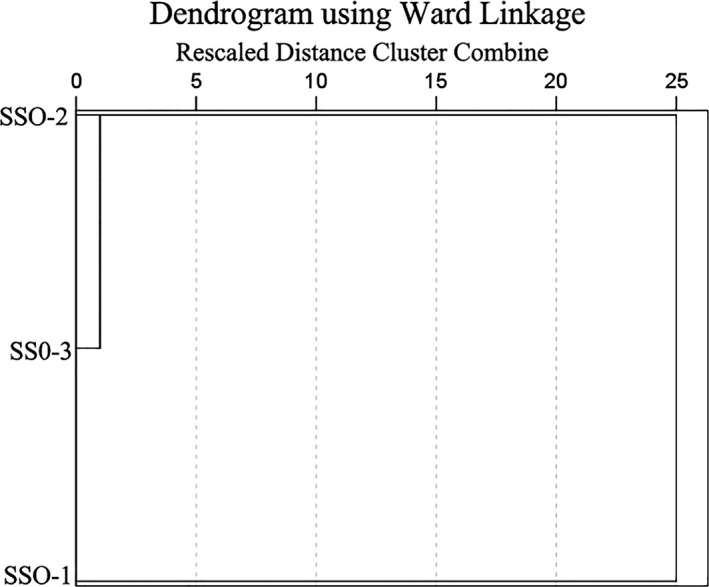
Hierarchical clustering analyses of aroma components of safflower seed oil from different regions

The different nature of volatile compounds prompted the need of a reliable analytical method that allowed their proper quantification to explain the method of panel test. In this study, all the volatile compounds extracted by HS‐SPME‐GC‐MS were analyzed by PCA to reduce the dimensionality of multivariate data. The score and loading plot of PCA are shown in Figure 3. 97.0% of variation in the data was explained by both PC‐1 (73.9%) and PC‐2 (23.1%), respectively (Figure 3a). SSO‐2 and SSO‐3 specimens were clustered together, while there was clear separation from SSO‐1. The major volatile compounds that positively correlated to PC‐1 were 2‐octene, n‐hexanal, acetic acid, and (Z)‐2‐heptenal, and those that positively correlated to PC‐2 were n‐hexanol, caproic acid (Figure 3b). Such clustering pattern of SSO was also revealed in hierarchical clustering analysis (Figure 4).

Hierarchical cluster analysis was further applied to creates groups of SSO based on their degree of association which reflected by distance between the samples. The results of HCA based on entire data of volatile components extracted by HS‐SPME‐GC‐MS were represented as dendrogram in Figure 4. Euclidean distance with Ward's linkage method was employed, and the distance between the samples was described by Euclidean in *y*‐axis. The SSO samples were divided into two groups, and in the first group, SSO‐2 and SSO‐3 were included while the second group included SSO‐1. SSO‐2 and SSO‐3 were closed, and these two samples were separated from SSO‐1.

## 4 DISCUSSION

### 4.1 Volatile compounds by HS‐SPME‐GC‐MS analysis

According to HS‐SPME‐GC‐MS analysis, there was a wide variety in proportion and composition of volatile compounds among different varieties of SSO as shown in Table 1. It is also the first time that n‐valeraldehyde, isoamyl alcohol, n‐pentanal, and heptanal were identified in SSO as aroma‐active components. 44, 41, and 47 volatile compounds were detected in SSO‐1, SSO‐2, and SSO‐3, respectively. This result related to cultivar, climate, and different environments of safflower. One hundred ninety‐three safflower accessions from forty countries were assessed for investigating the relationship between patterns of geographic diversity and agro‐morphological traits, showing that safflower seed diversity pattern related barely with DNA agricultural variety but geographic location (Khan, Witzke‐Ehbrecht, Maass, & Becker, 2008). Compared with our results, 77 volatiles of SSO from Turkey were quantified (Aydeniz et al., 2014), hexanal, benzyl alcohol, methyl benzene, heptanal, and 2‐octenal were identified with the highest frequency, in which aldehyde composition is similar to that of SSO from Xinjiang Autonomous Region, China, but the alcohols are quite different, which might be related to the high volatility of alcohols, and species diversity is presumably another important reason. However, Aydeniz et al. reported no ether compounds detected in SSO, and this result may be due to the ether residue considering extraction solvent in HS‐SPME; in addition, differences in variety, environment, and SPME coating are also considered to be the possibly main cause of conflict (Hashemi, Zohrabi, & Shamsipur, 2018).

All volatile compounds extracted were classified into nine categories (Figure 1), with alkene compounds (8.04%–22.77%), aldehydes (19.28%–44.75%), alcohols (9.16%–40.13%), acid compounds (9.89%–13.45%), and heterocyclic compounds (5.16%–16.92%) being relatively prevalent. It is noteworthy that aldehydes and alcohols were the predominant volatile compounds accounting for 28.44%–84.88% of aroma compounds detected in all of the three samples; n‐hexanal (10.61%–23.24%) and n‐hexanol (4.9%–29.93%) were the most abundant. These results much close to the information on volatile compounds of French and Spanish virgin olive oils reported that hexanal, (E)‐2‐hexenol, and hexanol to be the largest characterized components (Cavalli, Fernandez, Lizzani‐Cuvelier, & Loiseau, 2004). Aldehydes, mainly n‐hexanal, are dominating volatile compounds of cold‐pressed SSO, which is mainly produced by the oxidation of linoleic and oleic acid (Aydeniz et al., 2014). The high level of n‐hexanal may also closely contact with lipoxygenase pathway from polyunsaturated fatty acid, a previous study reported that linolenic and linoleic acids could produce n‐hexanal enzymatically through a reaction with 13‐hydroperoxide, and other aldehydes such as (Z)‐3‐hexenal and (Z)‐2‐hexenal were simultaneously formed as intermediate product of this reaction, which also play an important role in composition of aldehydes (Benincasa et al., 2003). Some researches indicated that aldehydes can be used as one of the oil quality management indicators, for example, (Z)‐2‐hexenal could partly explain quality differences in olive oils due to the increasing properties in postharvest storage (Kalua et al., 2007; Zheng et al., 2018). Alcohols also contributed significantly to volatile profile, of which n‐hexanol accounts for the largest proportion (4.90%–29.93%). Sánchez‐Ortiz, Pérez, and Sanz (2013) disclosed that alcohols were mainly produced by self‐oxidation pathway of fatty acids, while the enzyme route also represented a certain impact on it during the seed storage process. The relatively high level of n‐hexanol (4.90%–29.93%) also might be attributed to the activity of the lipid oxygenase and alcohol dehydrogenase enzyme (Sánchez‐Ortiz, Bejaoui, Quintero‐Flores, Jiménez, & Beltrán, 2018). This study provides the first data for comparing volatile compound composition of SSO of Xinjiang Autonomous Region, China.

### 4.2 Main odor‐active compounds

At present, most of the research on the aroma of vegetable oils is analyzed by HS‐SPME‐GC‐MS (Bueno, Resconi, Campo, Ferreira, & Escudero, 2019; Moreira et al., 2019). This method can only explain the chemical composition and content of volatiles in terms of chemistry, but not all volatile components contribute to the characteristic aroma of safflower seed oil. Vilanova et al. found that only 14 volatile compounds were finally revealed that contribute to aroma of Albariño wines despite 35 volatile quantification of GC analysis (Vilanova, Genisheva, Masa, & Oliveira, 2010). Similar to the results shown in Tables 1 and 2, only 16 aroma‐active compounds were selected though 67 volatile compounds were identified by HS‐SPME‐GC‐MS in our study. Although the individual proportions of the ingredients are low, they can also influence markedly the aroma and determine the overall flavor owing to their low sensory thresholds. β‐damascenone was observed to be the most active aroma compounds in Albariño wines due to its extremely low odor threshold of 0.05 ppb, despite with a low concentration of 0.90 μg/L (Vilanova et al., 2010).

Twelve key aroma components screened by ROAV > 1 from all volatile compounds through HS‐SPME‐GC‐MS were accounted by aldehydes, which were the largest group followed by alcohols (2), furan (1), and alkene compounds (1). Aldehydes, generally degradation products of fatty acids, were the most important key odor compounds with high peak area and low odor threshold (Genovese et al., 2018). Some studies also showed that there were certain correlations between C5‐C6 aldehydes and high‐quality vegetable oils (Kalua, Bedgood, Bishop, & Prenzler, 2013). It is known that grass, fatty, and fragrance were the major intensive flavors of SSO as shown in Table 2, in accordance with the grassy and fatty taste in the sensory panel, which might largely be related to flavor of certain aldehydes. (Z)‐2‐hexenal is often considered to be a volatile component in high‐quality olive oil with contributions to the characteristic flavor of sweet and vanilla (Veneziani et al., 2018). Nonanal was found at relatively high content (0.53%–1.51%) in three samples, which closely related to high linoleic acid in safflower seed oil. It is likely that in these samples, nonanal provides the highest contribution to the general odor of grass and almond, obtaining the ROAV of 98.23, 100.00, and 100.00, respectively (SSO‐1, SSO‐2, and SSO‐3). (Z)‐6‐nonenal also was a possible major odor compound providing a flavor of grass and fatty, which had a high contribution to the characteristic flavor with an extreme low odor threshold of 0.08 ppb in SSO‐1 and SSO‐3. Alcohols also produced an important fraction of volatile compounds of SSO from Xinjiang Autonomous Region. 1‐Octen‐3‐ol was previously reported to act as the primary flavor compound in mushroom (Deveci, Tel‐Cayan, Emin Duru, & Turkoglu, 2017). 1‐Octen‐3‐ol was found at minor relative concentration; nevertheless, it exhibited strong odor activity of mushroom and sweet having an odor threshold as low as 1 ppb and can influence markedly the aroma of SSO. N‐hexanol constituted an abundant content due to its high sensory threshold of 250 ppb and therefore is considered for a lesser extent of modification.

Although only 1 furan and 1 alkene were selected as key aroma‐active component with ROAV > 1, they also produced an essential fraction of volatiles. Limonene was a natural monoterpenoid compound. It is widely reported in lemon and orange products and also is major volatile component (ca 52% w/w of the total volatiles) in peel of Libyaone Libyan oranges (Macleod, Macleod, & Subramanian, 1988). Limonene, as the only alkenes selected, might be the only significant source of citrus flavor providing contribution to fruit and orange. As one of the heterocyclic compounds, furaneol has been identified previously as a product of the Maillard reaction (Zhang, Wang, Yuan, Yang, & Liu, 2016). 2‐N‐pentylfuran was perceived with a favorable paddy‐like note and has been intensively demonstrated to be the primary flavor compound in cereals (Multari et al., 2018). In a previous study about SSO, 2‐n‐pentylfuran was regarded as a key odor‐active compound and its role of the flavor has only been identified after seed roasting while cold‐pressed sample had none of it (Aydeniz et al., 2014). However, in this study, 2‐n‐pentylfuran was firstly indicated as the major odorant of cold‐pressed SSO of SSO‐2 variety. This was consistent with sensory panel.

Besides the components shown in Table 2, some potential odorants might also necessary to aroma profile but not detected by HS‐SPME‐GC‐MS. Though they represented at both low levels of threshold and concentration, these compounds may construct the delicate background aromas through interactions between each other (Yang et al., 2009). Thus, a further identification was needed to explore whether and how they contributed to the flavor.

### 4.3 Sensory evaluation of SSO

To obtain more intuitive sense result, a sensory evaluation was applied to reveal the further relationship between volatile components and sensory descriptors of different SSO (Figure 2). Apparently, all three samples were found to exhibit relative higher score in grass and fatty aroma than other sensory profiles, which could be regarded as basic flavor of SSO. In a previous study, Sakač et al. reported that aldehydes were generally described as grass and fatty aroma (Sakač et al., 2016). Most aldehydes including hexanal, (Z)‐6‐nonenal, (Z)‐2,4‐decadienal, and 2,4‐decadienal all showed positive impact on the contribution to grass and fatty aroma and made these two aroma profiles greatly strengthened. The almond odor was another basic flavor that had similar intensity among three samples, which perhaps is associated mainly with nonanal. Beltrán, Ramos, Grané, Martín, and Garrigós (2011) monitored the oxidative process of Spanish and American almond oils and indicated nonanal as a volatile compound that could be obtained in all samples.

SSO‐1 presented a statistically lower intensity (*p* < .05) of fruity odor (score = 6.1) than other two samples (8.1 and 8.2, respectively) as shown in Table 3. This result was probably correlated with lower content of limonene and octanal in SSO‐1; in a previous study, limonene and octanal also were reported widely in citrus fruit as major fruity aroma‐active compounds (López‐López et al., 2019). The significantly less intense (*p* < .05) of mushroom and sweet attribute in SSO‐1 might be explained by the lower content of 1‐octen‐3‐ol and 2‐octenal. Morales, Luna, and Aparicio (2005) studied the main sensory defects in virgin olive oil previously and indicated 1‐octen‐3‐ol, ethyl butanoate, propanoic, and butanoic acids evaluated by odor activity values, the most prominent volatile compounds responsible for mustiness–humidity, which is quite like the mushroom descriptor in our study. SSO‐3 gain highest score with 9.5 of the overall fragrance, which are responsible for the mixture of key aroma‐active compounds.

### 4.4 Statistical analysis

Principal component analysis is an unsupervised clustering technique that does not require knowledge of the data set and can reduce the dimension of multivariate data while maintaining uncertainty of the data (Farag, Weigend, Luebert, Brokamp, & Wessjohann, 2013). A clear discrimination among three samples (SSO‐2 and SSO‐3 were clustered together while separated from SSO‐1) was showed by PCA on the basis of all volatile components detected by HS‐SPME‐GC‐MS (Figure 3a). PCA was previously applied to the data detected from GC‐MS showing that variables such as sterol and triterpenic dialcohol allowed successful discriminating among the different varieties of Tunisian wild olive oils studied (Baccouri, Manai, Casas, Osorio, & Zarrouk, 2018). Taamalli et al. also regarded PCA as an effective tool to discriminate oil among different varieties and note a good discrimination between six Tunisian extra virgin olive oil according to components of pinoresinol acetate and elenolic acid. (Taamalli, Gómez‐Caravaca, Zarrouk, Segura‐Carretero, & Fernández‐Gutiérrez, 2010). Meanwhile, the loading plot (Figure 3b), which enabled us to visualize a possible relationship between the variety and volatile compounds, pointed out that volatile compounds including 2‐octene, n‐hexanal, acetic acid, (Z)‐2‐heptenal, n‐hexanol, and caproic acid significantly impacted on the discrimination. Furthermore, HCA was an algorithmic approach for constructing a hierarchy of clusters and further visualizes their relationship in dendrogram graphs (Cebi et al., 2017). The HCA result showed a cluster of SSO‐2 and SSO‐3 while separated from SSO‐1 (Figure 4); this clustering pattern consisted of PCA, as well as sensory analysis results above.

## 5 CONCLUSIONS

A comprehensive characterization of the aroma profile of SSO from Xinjiang Autonomous Region, China, was analyzed by HS‐SPME‐GC‐MS. There were 67 volatile substances tentatively identified in three samples. ROAV analysis further indicated that the flavor of SSO was more correlated with 12 aldehydes, two alcohols, one alkene, and one heterocyclic compounds according to cultivated varieties. Meanwhile, limonene, octanal, 2‐octenal, and 1‐octen‐3‐ol were major volatile compounds providing fruity, mushroom, and sweet aroma. The sensory variance analysis indicated that the odor profiles differed significantly with regard to the “fruity,” “mushroom,” “sweet,” and “paddy” aroma (*p* < .05), which largely in accordance with the key odor compounds. In addition, these aroma descriptions of SSO can play an essential role in the possibility of using volatile constituents to identify the source of safflower varieties. The result of PCA revealed a clear discrimination and demonstrated that PCA was a potential and useful tool for SSO primary evaluation of category similarity.

## CONFLICT OF INTEREST

There are no conflicts of interest.

## ETHICAL APPROVAL

This study does not involve any human or animal testing.
